# Mapping the Analytical Landscape of Gene–Diet Interactions in Epidemiology: From Classical Models to Causal and Multi-Omics Frameworks

**DOI:** 10.3390/nu18060880

**Published:** 2026-03-10

**Authors:** Andrea Maugeri

**Affiliations:** Department of Medical and Surgical Sciences and Advanced Technologies “GF Ingrassia”, University of Catania, 95127 Catania, Italy; andrea.maugeri@unict.it

**Keywords:** gene–diet interaction, nutritional epidemiology, regression models, study designs, dietary assessment, dietary patterns, genome-wide approaches, causal inference, multi-omics integration, machine learning

## Abstract

Diet is a major, modifiable determinant of cardiometabolic, cancer, and inflammatory disease risk, yet individuals frequently exhibit substantial heterogeneity in metabolic and clinical responses to similar dietary exposures. Genetic susceptibility and its interplay with diet plausibly contribute to this variability, motivating gene–diet (G×D) interaction research and the broader ambition of precision nutrition. Translation has lagged, however, because interaction effects are typically modest, context-dependent, and difficult to reproduce, particularly in the presence of pervasive dietary measurement error, heterogeneous exposure definitions, and stringent multiplicity correction. A methodologically oriented synthesis is presented across eight domains of contemporary G×D epidemiology: classical regression interaction models; efficient study designs; dietary assessment and measurement error; dietary patterns, mixtures, and non-linear modeling; genome-wide and polygenic approaches; causal inference frameworks; multi-omics integration; and machine learning. Central concepts include the recognition that “interaction” is a scale-dependent estimand and that transparent reporting of coding choices and effect-modification metrics—including additive interaction when relevant for public health interpretation—is essential. Credible inference further depends on high-quality, harmonized dietary phenotyping with explicit energy adjustment and, where feasible, biomarker calibration, alongside robust control of population structure and gene–diet correlation using ancestry adjustment, mixed models, and family-based designs. Genome-wide and polygenic risk-based approaches expand discovery potential but require disciplined multiplicity strategies, discovery-replication workflows, and explicit evaluation of portability and equity across ancestries. Causal inference methods can strengthen etiologic interpretation when assumptions are defensible and sensitivity analyses are routinely implemented. Multi-omics and machine learning may enhance mechanistic and predictive insight, but only under rigorous quality control, validation, and reproducible pipelines. Overall, harmonized measurement, clear estimands, multi-ancestry replication, and integrated evidence pipelines are pivotal for producing robust and actionable G×D evidence.

## 1. Introduction

Diet is a major, modifiable driver of cardiometabolic, cancer, and inflammatory disease risk. Yet people often show pronounced differences in metabolic and clinical responses even when their dietary exposures appear similar [1,2,3]. A meaningful portion of this variability likely reflects genetic susceptibility and its interplay with diet and other lifestyle factors, which underpins gene–environment (G×E) epidemiology and, more specifically, research on gene–diet (G×D) interactions [4,5].

The rationale for G×D research is closely linked to precision nutrition: the idea that tailoring dietary prevention and treatment to an individual’s biological profile—including genetic predisposition—could improve effectiveness, efficiency, and equity [6,7]. In practice, however, translation has been slower than expected. Interaction effects are often small, highly context-dependent, and difficult to replicate across populations, dietary assessment tools, and outcome definitions [1,5]. These issues are compounded by publication bias and by an evidence base that has historically been fragmented, with many studies focusing on single variants and single nutrients rather than on rigorous discovery-and-replication pipelines [4,8].

Early nutrigenetic research commonly relied on candidate-gene designs, testing a limited set of biologically plausible variants against specific nutrients or eating behaviors. Although attractive from a clinical perspective, such studies were frequently underpowered, burdened by multiple testing, and vulnerable to dietary misclassification, leading to inconsistent replication [4,5]. The field has increasingly moved toward biobank-scale resources with dense genotyping, improved imputation, and prospective follow-up, enabling genome-wide interaction studies (GEWIS) and polygenic approaches within transparent discovery-replication workflows [9,10,11]. Large, multi-country infrastructures illustrate how prospective designs and harmonized exposure measurements can support more reliable G×D inference at scale [12].

A further motivation for contemporary G×D work is the growing recognition that many dietary exposures are themselves partly heritable and may be affected by reporting biases that correlate with adiposity, socioeconomic position, and health status [13]. Genome-wide studies of diet-related traits in UK Biobank and other cohorts have identified hundreds of associated loci, but they also suggest that some genetic signals reflect downstream effects of traits such as body mass on food choices and on how diet is reported [13]. This highlights the need to separate etiologic effects of diet from genetic influences on dietary behavior and measurement, especially when interpreting G×D findings [14,15].

From a biostatistical perspective, “interaction” is not a single biological phenomenon but a model- and scale-dependent estimand describing effect modification on a chosen scale (e.g., multiplicative vs. additive) [16,17]. Because the interaction parameter depends on the link function and outcome scale, clear reporting of the estimand, scale, and coding of diet and genotype is essential [16]. Additive interaction measures are often more informative for public health planning than multiplicative interaction alone, yet they remain underreported in nutritional genomics [16,17].

Nutritional epidemiology adds complexity because dietary exposures are inherently multivariate and compositional. Total energy intake constrains macronutrient distributions, and increasing one dietary component typically requires decreasing another [18,19]. As a result, single-nutrient G×D models can be difficult to interpret unless they use energy-adjustment strategies, explicit isocaloric substitution contrasts, or higher-level representations of diet (patterns, mixtures, networks) that better reflect real-world eating behavior [18,20,21]. In addition, population structure and gene–diet correlation can bias interaction estimates if not addressed through ancestry adjustment, careful design, or within-family approaches [8,22].

In parallel, the analytical toolkit for G×D research has expanded beyond standard regression models with product terms. Modern studies increasingly use two-step genome-wide procedures, mixed-model variance component methods, efficient two-phase sampling, and causal inference frameworks. They also incorporate multi-omics data to move from association toward mechanism and causal interpretation [23,24,25,26,27,28]. Machine learning approaches can capture non-linearities and higher-order interactions, but they also raise challenges related to interpretability and reproducibility [29,30,31].

## 2. Analytical Landscape of Gene–Diet Interactions in Epidemiology

**Figure 1:**
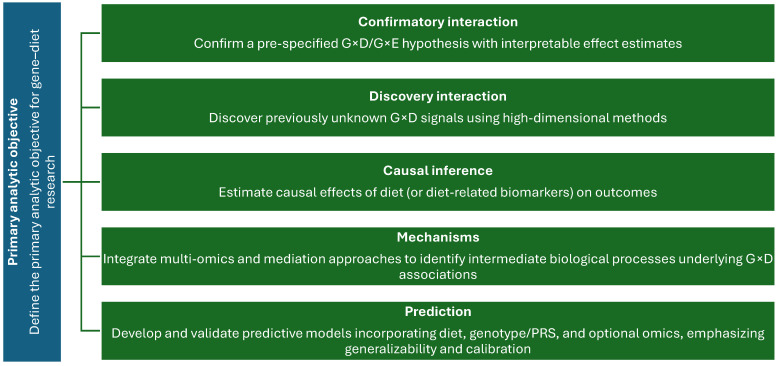
Core objectives of gene–diet (G×D) epidemiology.

Against this background, Figure 1 introduces the principal objectives of G×D research and motivates the need for a method-focused synthesis that maps the analytical landscape of G×D epidemiology, clarifies key assumptions and estimands, and provides practical guidance for transparent, reproducible implementation. In line with these aims, the present work organizes contemporary methods into eight domains: (1) classical statistical models; (2) efficient study designs; (3) dietary assessment and measurement error; (4) dietary patterns, mixtures, and non-linear models; (5) genome-wide, high-dimensional, and polygenic approaches; (6) causal inference frameworks; (7) multi-omics integration; and (8) machine learning. Whereas earlier nutrigenomics reviews have frequently focused on specific biological pathways or candidate-gene evidence, an updated methods-centered synthesis is increasingly warranted to reflect contemporary epidemiologic practice. This includes the widespread availability of large biobanks, the use of efficient two-phase and family-based designs, high-dimensional discovery strategies, and the growing integration of causal inference, multi-omics data, and machine learning within a unified analytical framework. The rapid expansion of scalable genotyping, imputation, and multi-omics resources has also shifted best practice toward multiplicity-aware discovery pipelines, explicit definition and reporting of interaction estimands, and reproducible, transparency-focused workflows. Accordingly, this review complements biology-oriented syntheses by providing a decision-oriented guide to the design, analysis, and interpretation of contemporary G×D studies [5,23,32,33].

### 2.1. Classical Statistical Models

Regression-based interaction models are still the most common way to test explicit G×D hypotheses in epidemiology [1,2,5]. In practice, they are implemented using generalized linear models (linear, logistic, Poisson) and time-to-event models (e.g., Cox regression), where the key feature is a product term between a genetic exposure (such as an SNP coded additively as 0/1/2) and a dietary exposure (nutrient intake, food groups, or diet-quality indices) [9,10]. This setup allows researchers to estimate how the genetic effect differs across levels of diet (or, conversely, how diet effects vary by genotype), and it yields parameters that are relatively straightforward to test, replicate, and meta-analyze [5].

Most G×D interaction studies are conducted within a frequentist framework, testing the null hypothesis using Wald, likelihood-ratio, or score tests, and reporting confidence intervals and *p*-values for the interaction coefficient (or for 2-degree-of-freedom joint tests of the genetic main effect plus interaction). In genome-wide applications, stringent multiplicity control is essential; commonly used approaches include family-wise error rate control (e.g., Bonferroni correction), false discovery rate (FDR) procedures, and q-values, often combined with two-step screening strategies to reduce the effective testing burden [23,34,35]. Bayesian approaches instead treat main and interaction effects as random variables and combine the likelihood with prior distributions, facilitating hierarchical shrinkage and principled uncertainty propagation across large numbers of correlated tests. In high-dimensional G×D scans, spike-and-slab specifications or global–local shrinkage priors (e.g., the horseshoe) can regularize the multitude of interaction parameters and yield posterior inclusion probabilities or posterior model probabilities as interpretable measures of evidence [36,37,38,39]. Bayes factors provide an alternative to *p*-values for ranking signals and can be calibrated to prior beliefs about plausible effect sizes in genetic epidemiology [40].

From a practical standpoint, frequentist testing remains attractive because it supports standardized, computationally efficient pipelines and straightforward meta-analysis. Bayesian modeling can be particularly advantageous when informative prior knowledge exists (e.g., candidate variants or pathway-based hypotheses), when effects are expected to be sparse, or when additional sources of uncertainty must be modeled explicitly (e.g., dietary measurement error or omics-derived exposures) [39,41,42]. Because Bayesian results can be sensitive to prior choices and computational implementation, transparent reporting should document prior specifications, posterior summaries (including credible intervals, which differ conceptually from confidence intervals), convergence and model-fit diagnostics, calibration checks where appropriate, and sensitivity analyses to alternative priors or model forms [36,43]. Importantly, Bayesian modeling does not automatically eliminate the multiple-testing problem in genome-wide or high-dimensional interaction scans. Rather, it shifts the burden to explicit prior structure, hierarchical modeling, and calibrated decision rules.

Building on these inferential considerations—whether frequentist or Bayesian—credible G×D estimation still hinges on correct model specification at the data level. Correct implementation depends on careful choices about variable definition and coding, as well as covariate adjustment. Genetic variants are often coded as effect-allele counts, though dominant or recessive models can sometimes be biologically justified [9]. Dietary variables may be modeled continuously (often per standard deviation), categorically (e.g., quintiles), or as summary scores for dietary patterns; these decisions directly affect statistical power and interpretability [18,20]. In cohort and biobank data, controlling confounding and population structure is essential: ancestry principal components are routinely included to reduce bias from population stratification, and sensitivity analyses (e.g., restricting to more homogeneous ancestry groups) are often recommended [22,32].

Large-scale datasets introduce additional technical and design issues. Analyses commonly adjust for study center, genotyping array, and other technical covariates; where possible, mixed models or within-family designs can further reduce bias due to cryptic relatedness or residual structure [24,44]. “Genomic control” methods may help detect or reduce test statistic inflation in genome-wide scans, but they cannot replace appropriate modeling of ancestry and relatedness [22,45].

Interaction can be tested using either a 1-degree-of-freedom (1-df) test focused on the interaction coefficient, or a 2-degree-of-freedom (2-df) joint test of the genetic main effect plus interaction [9,46]. The 2-df approach can be more powerful for discovery because it can flag loci with either a main effect or an interaction effect, but it can complicate interpretation and replication unless results are clearly decomposed and reported [46]. Another central issue is the scale of interaction. Standard regression outputs typically reflect multiplicative interaction (e.g., on the odds ratio or hazard ratio scale), whereas additive interaction (e.g., relative excess risk due to interaction, attributable proportion) is often more informative for public health impact and synergy [16,17]. Reporting both scales—or explicitly justifying the chosen scale—is therefore advisable [16].

Despite their clarity, classical models face challenges that are particularly acute in dietary research. Measurement error in diet tends to attenuate interaction effects and inflate uncertainty, making modest G×D effects hard to detect even in large samples [41,47]. Residual confounding—for example by socioeconomic status, health consciousness, or correlated lifestyle factors—can also create spurious effect modification if diet is imprecisely measured or key confounders are not fully captured [33,48]. Finally, diet-outcome relations are often non-linear; if non-linearity is ignored (e.g., not using splines), apparent interaction signals may reflect model mis-specification rather than true biological effect modification [21].

Applied studies illustrate both strengths and limitations of this framework. EPIC-InterAct combined systematic review evidence with harmonized, prospective analyses of gene–macronutrient interactions and incident type 2 diabetes across several European cohorts, showing pragmatic solutions for dietary harmonization and interaction testing in survival models [12]. In U.S. cohorts (Nurses’ Health Study; Health Professionals Follow-up Study), adherence to healthier dietary patterns was reported to attenuate associations between BMI-related polygenic risk and long-term weight gain, demonstrating how pattern-based exposures can be used in longitudinal interaction models [11]. Large biobank analyses have similarly examined whether diet quality modifies genetic associations with glycemic traits such as HbA1c, providing examples of G×D interaction at population scale [10].

### 2.2. Efficient Study Designs

Efficient designs seek to increase statistical power and feasibility when it is impractical to measure genotypes, detailed diet, biomarkers, or omics in every cohort participant [4,5]. This is especially relevant for expensive components such as repeated dietary recalls, objective dietary biomarkers, tissue-specific omics profiling, and long-term prospective follow-up [28,48,49,50]. The core idea is to allocate intensive measurements to the most informative individuals or strata, while applying design-aware analysis to retain unbiased population-level inference [5].

A well-known example is the case-only design, which can estimate interaction efficiently because—if gene and diet are independent in the source population—the association between genotype and diet among cases identifies the interaction parameter [4,51]. In nutritional genomics, however, the independence assumption is often doubtful. Gene–diet correlation can arise because genetics influences appetite, taste, adiposity, or health-related behaviors, and population stratification can also generate spurious gene–diet associations [8,13]. When independence is violated, case-only estimates can be substantially biased. Therefore, such analyses require supporting evidence (e.g., testing gene–diet association in controls, sensitivity analyses, or negative-control strategies) [51].

More robust alternatives include nested case–control and case–cohort designs, which preserve the prospective cohort structure but reduce costs by genotyping or profiling only a subset [5]. A broader framework is two-phase sampling: inexpensive variables are collected for all participants in phase I, while expensive genotyping, deep dietary measures, or omics data are collected in a strategically selected phase II subsample (often enriched for outcome cases or exposure extremes) [5]. Valid inference then requires estimators that explicitly account for sampling—such as inverse probability weighting, pseudo-likelihood, or full likelihood approaches—and variance estimates that reflect the design [5].

Family-based and within-sibship designs can further strengthen robustness when gene–diet correlation or population stratification is a concern, because comparisons within families naturally control for many shared background factors [8,44]. For instance, a family-based study in Northern China assessing fruit intake and CMIP rs2925979 in relation to type 2 diabetes illustrates clustered-data approaches to diet–gene interaction [44]. In inflammatory disease contexts, reviews have similarly emphasized that rigorous design and bias control are crucial when diet is complex and potentially genetically correlated [51].

Across efficient designs, transparent reporting is essential. Studies should describe sampling frames, phase-II selection probabilities or rules, participation rates, missingness and its likely mechanisms, and the analysis method used to correct for sampling (weights or likelihood) [33]. For genetic interaction studies, reporting should also align with STREGA recommendations, including documentation of ancestry adjustment, relatedness handling, and genotyping/imputation quality control [32].

### 2.3. Dietary Assessment and Measurement Error

The quality of dietary assessment is a key driver of both validity and statistical power in G×D research, because interaction effects are often small and highly sensitive to exposure misclassification [5,41]. Common instruments include food-frequency questionnaires (FFQs), repeated 24 h recalls, food diaries/records, and—when feasible—objective biomarkers (e.g., recovery biomarkers for energy and protein, and concentration biomarkers for selected micronutrients) [48,49,50]. These tools differ in their error profiles. FFQs may better reflect longer-term habits but often suffer from systematic bias and limited detail, whereas multiple 24 h recalls can capture day-to-day variability but require repetition to estimate usual intake reliably [50,52].

Measurement error typically attenuates regression coefficients and reduces power, but its impact on interaction terms can be even more damaging, because detecting interaction requires identifying differences in slopes across strata [5,41]. Validation studies have documented substantial random and systematic error for key dietary components, highlighting why modest G×D effects are difficult to detect using self-reported diet alone [47]. Moreover, differential error—for instance, changes in reporting after diagnosis, or genotype-related differences in reporting mediated by adiposity—can bias interaction estimates away from the null and generate spurious signals [13,41].

Generalizability Theory (G-theory) offers a useful framework for formalizing the reliability of dietary assessment in multi-facet designs (e.g., persons × days × interviewers × seasons) by decomposing total variability into variance components attributable to each facet and their interactions [53,54]. In a generalizability study, variance-component estimates quantify the extent to which measurement error arises from within-person day-to-day variation versus other sources. In a decision study, these components inform efficient data-collection planning (e.g., the number and timing of 24 h recalls, balancing weekdays and weekends, or prioritizing a calibration sub-study) to achieve a prespecified generalizability or dependability coefficient. Empirical applications to repeated 24 h dietary recalls indicate that within-person variability is often a dominant contributor for many nutrients, implying that multiple repeated recalls may be required to estimate usual intake with acceptable precision [55,56].

Several approaches can reduce or account for dietary measurement error. Regression calibration and related measurement-error models use validation or biomarker data to correct attenuation. These methods can be extended to interaction settings but require explicit assumptions about the error structure and careful modeling when error depends on covariates [41,42]. When repeated recalls are available, mixed-effects models for usual intake can reduce within-person random error, improve exposure precision, and potentially increase power to detect interaction [52]. In addition, sensitivity analyses and quantitative bias analysis can be used to evaluate how robust interaction findings are to plausible levels of misclassification and residual confounding [41].

For interaction analyses, it is useful to state the assumed measurement-error model explicitly. Many correction methods implicitly assume approximately classical, nondifferential error in dietary exposure—namely, that the observed intake equals the true (latent) intake plus random error that is independent of the outcome and, critically, does not vary by genotype beyond what is explained by measured covariates. Under departures from these assumptions, inference can change substantially: systematic error and differential error by outcome status or genotype can bias interaction estimates in either direction and may generate spurious effect modification when none is present. Accordingly, the assumed error structure should be documented transparently, and structured sensitivity analyses should be conducted to evaluate the robustness of G×D findings to plausible violations of classical assumptions [41,42].

Energy adjustment is foundational in nutritional epidemiology and is particularly important in G×D analyses of nutrients, because total energy intake is associated with many outcomes and strongly correlates with nutrient intakes [18]. Methods such as the residual approach and nutrient density models help deliver isocaloric interpretations and reduce confounding by total energy [18]. When the etiologic question concerns replacing one macronutrient with another, explicit isocaloric substitution models are essential. In interaction analyses, substitution contrasts can be interacted with genetic predictors to test whether the health effect of macronutrient replacement differs by genotype [18,19]. Given the compositional nature of macronutrients, compositional data methods can provide more coherent interpretations and may reduce artifacts from collinearity and unit-sum constraints [19].

Finally, dietary exposure construction should be reported in enough detail to allow replication: the assessment instrument, time window, number of repeated administrations, energy-adjustment method, handling of misreporting/outliers, and derivation of composite scores [33,48]. In G×D studies these details are not minor, they often determine whether interaction results are interpretable and reproducible [5].

### 2.4. Dietary Patterns, Mixture Models, and Non-Linear Methods

Because foods and nutrients are consumed together and are biologically interdependent, representing diet as a multidimensional exposure—rather than as isolated nutrients—has become increasingly important in G×D research [21,57]. Dietary pattern approaches include a priori indices (based on guidelines or prior evidence) and data-driven methods such as principal component analysis (PCA), factor analysis, and clustering, which summarize co-consumption into latent patterns [20,21]. In G×D studies, pattern scores can be interacted with SNPs to test whether higher diet quality mitigates genetic susceptibility. An advantage is that pattern-based results often translate more directly into actionable dietary recommendations [10,11].

Reduced rank regression (RRR) and related partial least squares methods extend pattern analysis by using intermediate response variables (e.g., lipids, inflammatory markers, metabolomic factors) to derive patterns more closely tied to hypothesized biological pathways [20,21]. In nutrigenomic applications, RRR can be useful for mechanistic questions—such as whether genetic effects differ in dietary contexts that influence proximal intermediates—but it depends heavily on the choice and measurement quality of response variables and requires strong internal validation to limit overfitting [21].

Methods for dietary mixtures, often adopted from environmental epidemiology, address collinearity and joint exposure effects among dietary components. Weighted quantile sum (WQS) regression constructs a mixture index and component weights under assumptions about directional effects, providing a parsimonious summary when many dietary factors are correlated [58]. Quantile g-computation relaxes some constraints by allowing components to contribute in different directions and yields an overall mixture effect interpretable as a joint shift in all components [59]. Bayesian kernel machine regression (BKMR) offers flexible modeling of non-linear and non-additive mixture effects and can be extended to explore effect modification by genotype by including gene terms and gene–mixture interaction components [60]. These approaches align well with real-world dietary change (multiple components shift together), but they increase model complexity and require careful pre-specification, tuning, and validation [20,21].

Non-linear modeling is often essential in nutrition because dose–response relations may involve thresholds, plateaus, or U-shaped associations [21]. Spline-based models and generalized additive models (GAMs) can capture such non-linearities and, when paired with genetic predictors, can describe genotype-specific dose–response curves that might be missed under linear assumptions [21]. Network analysis provides another representation by modeling foods or nutrients as nodes and co-consumption as edges. Recent guidance stresses standardized network construction, sensitivity analyses, and transparent reporting of network metrics to improve reproducibility [61].

Across pattern, mixture, and non-linear approaches, implementation hinges on: (i) transparent exposure construction (food grouping, scaling, energy adjustment); (ii) internal validation (split-sample checks, bootstrapping, stability metrics); (iii) external replication across cohorts with comparable dietary instruments; and (iv) translating outputs into interpretable, actionable dietary constructs [20,21,61]. Without these steps, added flexibility can increase overfitting and reduce portability across populations.

### 2.5. Genome-Wide, High-Dimensional, and Polygenic Approaches

Genome-wide strategies aim to identify genetic loci whose associations with health outcomes depend on diet, moving beyond pre-specified candidate genes to hypothesis-free scans across millions of variants [9,62]. In a typical GEWIS, models include the main effects of genotype and diet plus their interaction term, while applying stringent control of type I error to address the large multiple-testing burden [9]. Because interaction effects are usually modest and dietary measurement error is common, GEWIS generally requires very large sample sizes and well-characterized dietary exposures to achieve adequate power [5,41].

To improve efficiency, many studies use two-step or screening-based procedures. These approaches first prioritize variants (e.g., based on marginal genetic associations or other filters) and then test interactions in a reduced set, lowering the effective multiple-testing burden while maintaining false-positive control under specific conditions [9,23]. Recent work also emphasizes methods that scale to biobank data and remain robust in the presence of relatedness and heterogeneity, including mixed-model frameworks [24,25,63]. A complementary perspective treats interaction signals as forms of context-specific heritability, enabling alternative tests that can be more powerful under certain genetic architectures [25].

Irrespective of the inferential paradigm, GEWIS requires a clearly specified strategy for controlling multiplicity—such as genome-wide significance thresholds, FDR/q-value procedures, or Bayesian priors that induce multiplicity adjustment—together with transparent reporting of the discovery-replication architecture, including whether interaction testing was conducted in a single-stage genome-wide scan or implemented within a two-stage (screening and testing) design [23,34,35,36,40,64].

Applied studies illustrate both promise and ongoing constraints. For example, a genome-wide interaction analysis of fiber, fruit, and vegetable intake in relation to colorectal cancer risk demonstrates that GEWIS is feasible for complex dietary exposures, but also highlights the need for harmonized exposure definitions and very large samples [65]. In metabolic outcomes, biobank-scale studies of macronutrient intake interacting with genetic variation for glycemic traits similarly show the scale required to detect small effects and the importance of careful control for confounding and measurement issues [66]. Population-specific investigations (e.g., in the Korean Genome and Epidemiology Study) provide additional SNP-level examples for cardiovascular outcomes, underscoring both the value of ancestry-specific discovery and the challenges of generalizing results across populations [67].

A related, aggregation-based strategy uses polygenic risk scores (PRS). PRS×diet models test whether diet modifies the association between a PRS and an outcome, potentially improving power by collapsing many variants into a single predictor [10,11]. These models are attractive for prevention and risk stratification, but their validity depends on how the PRS is built, how well it is calibrated, and whether it transfers across ancestries—an area where portability remains a major concern [68,69]. There is also an equity issue: PRS-guided dietary recommendations could widen disparities if PRS are derived from non-representative datasets or if the resulting interventions are not equally accessible [69]. For these reasons, transparent reporting of PRS derivation, validation, and calibration is essential, consistent with guidance for genetic risk prediction studies [70].

### 2.6. Causal Inference Frameworks

Causal inference approaches aim to strengthen etiologic interpretation in G×D research by addressing confounding, reverse causation, and mechanistic pathway questions [15,26]. A widely used tool is Mendelian randomization (MR), which treats genetic variants as instrumental variables for diet-related traits or biomarkers, leveraging the quasi-random allocation of alleles to reduce confounding—provided core assumptions hold (relevance, independence, exclusion restriction) [26]. In nutrition, MR faces practical obstacles: for many dietary exposures, strong and specific genetic instruments are scarce, and horizontal pleiotropy (variants affecting outcomes through pathways other than the exposure) can bias estimates [14,15].

To improve robustness, MR studies increasingly use sensitivity analyses and alternative estimators. MR-Egger can provide pleiotropy-robust estimation under an additional “InSIDE” assumption, while mixture-model and related approaches can down-weight or explicitly model invalid instruments [71,72]. Outlier-based procedures such as MR-PRESSO, along with robust estimators suited to many instruments, further support inference when some instruments violate assumptions [73,74]. Multivariable MR can estimate causal effects of correlated exposures (e.g., multiple nutrients or biomarkers), but requires careful assessment of conditional instrument strength and interpretation under potentially complex pleiotropy [75]. Three-sample designs have also been proposed to reduce bias and improve robustness in summary-data MR settings [76].

Large-scale MR applications have scanned many dietary habits for potential causal links with cardiovascular outcomes, illustrating both the opportunity of hypothesis-wide MR and the limitations imposed by instrument quality and pleiotropy [77,78]. Importantly, MR is usually not a direct test of effect modification. Instead, it can complement G×D interaction studies through triangulation, stratified MR, or by testing whether diet-related intermediates (e.g., biomarkers) plausibly lie on pathways that could mediate G×D effects [14,15].

Beyond MR, longitudinal causal methods such as g-methods and targeted maximum likelihood estimation (TMLE) address time-varying confounding and dynamic exposures, common features of diet research where diet, weight, and medication use evolve over time [27]. By explicitly modeling exposure and censoring processes and targeting well-defined causal estimands, these approaches can estimate the effects of sustained dietary patterns or interventions under assumptions such as exchangeability and positivity. They can also incorporate machine learning for nuisance functions while retaining valid inference under double robustness [27]. Finally, causal mediation analysis can quantify how much of an effect operates through specific intermediates (including molecular mediators), but it requires strong identification assumptions and careful handling of mediator–outcome confounding [79,80].

### 2.7. Multi-Omics Integration

Multi-omics integration expands G×D research by connecting genetic and dietary exposures to intermediate molecular phenotypes, which can help clarify biological pathways and make interaction findings more interpretable [28,81]. Common omics layers include epigenomics (e.g., DNA methylation), transcriptomics, proteomics, metabolomics/lipidomics, and the gut microbiome. Each layer brings distinct analytical challenges—batch effects, sparsity, missing data, and severe multiple-testing burdens—so careful quality control, normalization, and sensitivity analyses are prerequisites for credible inference [28].

A range of integrative methods is used to combine information across omics blocks. These include latent factor models (e.g., Multi-Omics Factor Analysis), integrative clustering (iCluster), supervised multi-block approaches (DIABLO), and network/module discovery methods such as WGCNA [28,82,83,84]. Such tools can support: (i) hypothesis generation (identifying molecular signatures jointly related to diet and genetic risk); (ii) mediation analyses (quantifying how much of an effect operates through a molecular pathway); and (iii) interaction-focused analyses (testing whether diet modifies genetic effects on omics signatures, or whether omics endophenotypes mediate observed G×D associations) [80].

Multi-omics datasets are frequently high-dimensional, with the number of measured features substantially exceeding the number of individuals, and are further shaped by pervasive correlation among features and platform-specific batch effects, making overfitting a primary threat to validity. Best practice therefore pairs dimension reduction and/or regularization with rigorous preprocessing—normalization, batch correction, feature filtering, and harmonized annotation—and prioritizes replication in independent cohorts and, where feasible, cross-platform validation of key signals. When multiple omics layers are analyzed jointly, the multiplicity strategy should be specified explicitly (e.g., layer-specific FDR control and/or hierarchical testing) to limit false discoveries [34,35,81,82,83,84].

Applied work illustrates both potential and constraints. The BarcUVa-Seq study, for example, linked dietary exposures to gene expression in healthy human colon tissue, showing how tissue-specific omics can provide mechanistic insight, while also emphasizing the dependence on high-quality dietary phenotyping [81]. Disease-oriented pipelines have integrated machine learning, MR, and mediation to prioritize candidate genes and pathways (e.g., in diabetic nephropathy), reflecting a broader convergence between systems biology and causal inference in modern G×D research [85]. At the same time, many multi-omics studies remain limited by sample size relative to feature dimensionality, which increases the risk of false discovery. Therefore, strong internal validation and independent replication are essential for generalizability [28,83].

Integrative multi-omics workflows sometimes treat molecular features as mediators linking diet and genotype to disease outcomes. However, mediation analysis supports causal interpretation only under strong identification conditions, including adequate control of confounding for the exposure–mediator, mediator–outcome, and exposure–outcome relationships, as well as correct model specification. In observational multi-omics settings, mediation findings should therefore be framed primarily as hypothesis-generating, unless strengthened by designs that better support causal inference (e.g., randomized dietary interventions, longitudinal g-methods for time-varying processes, or valid genetic instruments for the proposed mediators) [79,80].

### 2.8. Machine Learning

Machine learning provides flexible tools for modeling non-linear relationships and high-order interactions among diet, genetic variation, omics features, and clinical outcomes—situations where standard parametric models may be misspecified [27,29]. Common supervised approaches include random forests, gradient boosting machines, and Bayesian additive regression trees (BART), which can capture complex interactions implicitly. Interaction-focused techniques (e.g., multifactor dimensionality reduction) have also been used, especially in genetic contexts [86,87,88,89]. In nutrition research, machine learning is additionally applied to derive dietary patterns or subtypes from high-dimensional intake data and to predict outcomes using integrated diet-omics profiles [29,90]. Importantly, many machine-learning applications are optimized for prediction rather than etiologic inference: a model can achieve high predictive performance by exploiting correlations (including confounding and selection artifacts) without identifying causal effect modification. When the goal is etiologic G×D inference, analysts should pre-specify the target estimand, use designs and adjustment strategies aligned with the causal structure, and separate discovery from confirmation via external validation or pre-registered replication.

These methods come with important risks. Machine learning can inadvertently learn confounding structure, batch effects, or selection artifacts, producing apparently strong “interaction” signals that fail to generalize [27,29]. For this reason, rigorous validation—nested cross-validation, calibration checks, and, ideally, external replication—is critical, along with strict separation between training and test data [30,31]. When the objective is explanation rather than prediction, interpretability methods—such as feature importance, partial dependence, and SHAP-like summaries—can support hypothesis generation, but they should be interpreted cautiously and, where possible, evaluated in independent datasets or embedded within causal inference frameworks [27,91]. These tools primarily describe the behavior of a fitted model and can be unstable under resampling, while also becoming misleading in the presence of correlated predictors or differences in scaling—features that are common in dietary, genomic, and multi-omics data. Standard random-forest importance measures may be biased, and Shapley-based explanations can be distorted when features are statistically dependent [92,93,94]. Accordingly, reporting should include stability assessments (e.g., bootstrap or repeated cross-validation), sensitivity analyses to preprocessing and feature engineering choices, and an explicit distinction between explaining model predictions and inferring biological mechanisms.

Finally, machine learning-based G×D studies should be reported with the same methodological transparency expected of classical analyses: clear objectives (prediction vs. etiologic inference), reproducible preprocessing and feature-selection steps, and well-documented pipelines [30,33]. For the genetic components, adherence to STREGA and related reporting standards remains important so that essential details—ancestry adjustment, genotyping/imputation quality control, and multiplicity control—are not obscured by model complexity [32].

## 3. Discussion

The analytical landscape of G×D epidemiology has broadened substantially, evolving from classical regression-based interaction testing toward a larger methodological ecosystem that now includes efficient sampling designs, genome-wide interaction scans, mixed-model and variance-component frameworks, causal inference tools, multi-omics integration, and machine learning. Yet, despite this diversification, the field still operates largely in a small-effect regime. Most interaction effects are modest, and even genuinely causal signals can be obscured by dietary measurement error, residual confounding, population structure, and stringent multiplicity correction. In this context, replication, triangulation, and transparent reporting are not optional add-ons, they are the core conditions for credibility [4,5,41].

A consistent message across paradigms is that the strength of G×D inference is often determined less by sophisticated modeling than by the quality and comparability of dietary phenotyping. Diet is difficult to measure with precision, and misclassification attenuates effects while inflating uncertainty—problems that become even more severe for interaction terms, which essentially require detecting differences in slopes across exposure strata [41]. This is why harmonization of dietary measures across cohorts, explicit energy adjustment, and—where feasible—calibration using objective biomarkers remain foundational [18,47]. Importantly, these decisions influence not only statistical power but also interpretation: without clear energy-adjusted and isocaloric contrasts, a “nutrient interaction” can be difficult to translate into a real-world dietary recommendation [18].

Equally central is conceptual clarity about what “interaction” means. In epidemiologic practice, interaction is a scale-dependent estimand rather than a singular biological entity, and conclusions can change depending on whether effect modification is assessed on multiplicative or additive scales [16]. Additive interaction measures often align more directly with public health relevance—capturing whether combined exposures generate excess absolute risk beyond what would be expected from each exposure alone—yet they remain underreported [16]. A practical implication is that future G×D studies should more routinely report both multiplicative and additive interaction—or clearly justify the chosen scale—with explicit documentation of genotype and diet coding, to support consistent interpretation and downstream synthesis [16].

Population structure and gene–diet correlation represent another set of issues that are especially salient in nutrigenomics. Genetic stratification can create spurious associations, and gene–diet correlation may arise because genetic variation influences appetite, adiposity, taste preferences, or health-related behaviors—factors that also shape diet and diet reporting [22,23,24]. Robust strategies therefore include careful ancestry adjustment, mixed-model approaches, and, when possible, family-based or within-sibship designs that reduce confounding from shared background factors [22,23,24]. These safeguards are not merely technical; they determine whether an apparent interaction reflects biology, behavior, measurement, or structure.

Conceptually, observed gene–diet correlation may reflect at least three distinct mechanisms, each with different analytic implications: (i) confounding by ancestry or broader social and environmental structure that influences both genotype frequencies and dietary behaviors; (ii) genuine gene–environment correlation, whereby genotype affects diet through causal pathways such as taste perception, appetite regulation, adiposity, or health-related behaviors; and (iii) collider or selection bias arising when analyses condition on variables—including study participation, disease status, or diet-related behaviors—that are jointly influenced by genotype and other determinants of diet or the outcome. Distinguishing among these mechanisms motivates routine assessment of gene–diet associations in the source population (or among controls), rigorous ancestry adjustment, and cautious interpretation of case-only or stratified analyses [95].

At the high-dimensional end of the spectrum, genome-wide and polygenic approaches have increased discovery potential, but they also sharpen the need for disciplined study architecture. Multiplicity correction is unavoidable, and in the presence of small effects and noisy exposures, false positives and unstable estimates become major threats unless discovery-replication workflows and pre-specified analysis plans are standard [4,5,41]. PRS adds a pragmatic layer: PRS×diet interactions can improve power by aggregating signals across variants and are appealing for prevention stratification. However, they carry two critical obligations: (i) explicit evaluation of PRS performance and calibration across ancestries, and (ii) attention to the equity implications of using PRS to allocate dietary interventions or counseling resources. If PRS are developed primarily in non-representative datasets, portability limitations can systematically disadvantage underrepresented groups; if diet interventions are not equally accessible, PRS-guided recommendations may inadvertently widen disparities [68,69,70].

Causal inference frameworks offer a complementary route to strengthen etiologic interpretation, but they also require careful humility about assumptions. MR can reduce confounding and reverse causation for certain diet-related traits and biomarkers, but nutritional applications often face weak or non-specific instruments and pervasive pleiotropy [26,71,73,75]. Modern longitudinal approaches, including g-methods and TMLE, better reflect the reality that diet and confounders (weight, medications, comorbidity) change over time, yet they demand strong assumptions and careful sensitivity analyses [27]. In practice, the most persuasive causal arguments in G×D research are likely to come from triangulation, converging evidence from classical interaction models, genetically informed designs, robust sensitivity analyses, and complementary causal methods rather than reliance on a single analytic paradigm [26,27,71,73,75].

Multi-omics integration and machine learning expand the field’s mechanistic and predictive horizons but introduce new fragilities. Multi-omics data can clarify pathways by linking diet and genotype to intermediate molecular phenotypes, improving biological interpretability and identifying plausible mediators [28,83]. However, omics layers are high-dimensional and sensitive to batch effects, missingness, and preprocessing decisions; without rigorous quality control and independent replication, they can amplify false discovery [28,83]. Similarly, machine learning can capture non-linearities and complex interactions that classical models miss, but it can also learn confounding structure or technical artifacts, producing “interaction-like” patterns that do not generalize. Robust validation, strict train-test separation, and transparent pipelines are therefore essential if machine learning outputs are to contribute to etiologic inference rather than only prediction [30,31].

Taken together, these considerations point toward a practical vision for next-generation G×D epidemiology: a coherent evidence pipeline built on harmonized measurement, explicit estimand definition, and multi-stage validation [32,33]. Methodologically, treating diet as a structured, multivariate exposure—and aligning analytical choices with clearly stated causal questions—should reduce ambiguity and improve translation into actionable guidance [16,18]. Second, stronger attention to study design for bias control can improve credibility. Mixed models and within-family analyses can reduce confounding from structure and shared background factors, while multi-cohort harmonization efforts can enable consistent discovery–replication pipelines across diverse settings [22,23,24,32,33]. Importantly, expanding multi-ancestry analyses is not only a generalizability goal but also a scientific necessity for evaluating portability and equity, especially for PRS-guided stratification [68,69,70]. Third, future work will benefit from more deliberate integration of causal inference into G×D questions. Rather than treating interaction tests as endpoints, studies can use triangulation—combining classical interaction models with MR and longitudinal causal methods when assumptions are plausible—to strengthen etiologic interpretation and to test pathway hypotheses under complementary sources of bias [26,27,71,73,75]. Clear reporting of estimands (including additive interaction where relevant) will be critical for synthesizing evidence and assessing public health impact [16]. Finally, multi-omics and machine learning can move the field beyond “does it interact?” toward “through what mechanism and for whom?”. Multi-omics can identify intermediate molecular signatures that sharpen biological plausibility and highlight actionable pathways, while machine learning can support risk prediction and subgroup identification—provided that rigorous quality control, validation, calibration, and transparent pipelines are standard practice [28,30,31,83]. Combining these tools within reproducible workflows can help convert high-dimensional discovery into robust, interpretable, and clinically relevant insight [32,33].

## 4. Conclusions

G×D epidemiology has matured into a broad methodological enterprise spanning classical interaction models, efficient designs, genome-wide and polygenic approaches, causal inference, multi-omics integration, and machine learning. Yet the field’s main challenge remains unchanged: most interaction effects are modest and highly sensitive to measurement error, structure, and multiplicity, making replication and triangulation foundational. The most credible path forward is a coherent evidence pipeline that prioritizes (i) high-quality, harmonized dietary phenotyping with explicit energy adjustment and, where feasible, calibration; (ii) clear definition and reporting of interaction estimands and scales; (iii) rigorous control of population stratification and gene–diet correlation; and (iv) transparent, pre-specified high-dimensional workflows with appropriate multiplicity strategies. Translation efforts—especially those involving PRS-based stratification—must explicitly address portability across ancestries and equity implications. When combined with causal inference and validated multi-omics/machine learning workflows, this pipeline can shift G×D research from fragile associations toward reproducible, interpretable findings that more plausibly inform precision nutrition and population health.

## Data Availability

No new data were created or analyzed in this study. Data sharing is not applicable to this article.
